# Glutamate/GABA/glutamine ratios in intact and ischemia reperfusion challenged rat brain subregions, the effect of ischemic preconditioning

**DOI:** 10.1007/s11011-024-01511-8

**Published:** 2025-02-07

**Authors:** Eva Baranovicova, Dagmar Kalenska, Jan Lehotsky

**Affiliations:** 1 Biomedical Centre Martin, Jessenius Faculty of Medicine in Martin, Comenius University Bratislava, Mala Hora 4, 036 01 Martin, Slovakia; 2 Department of Anatomy, Jessenius Faculty of Medicine in Martin, Comenius University Bratislava, Mala Hora 4, 036 01 Martin, Slovakia; 3 Department of Medical Biochemistry, Jessenius Faculty of Medicine in Martin, Comenius University Bratislava, Mala Hora 4, 036 01 Martin, Slovakia

**Keywords:** Rat, Cerebral ischemia, NMR metabolomics, Glutamate, Glutamine, GABA, Ischemic preconditioning

## Abstract

The proper function of the brain is entirely dependent on intact neurotransmission, where glutamate (Glu) and γ-aminobutyric acid (GABA) are the two most present neurotransmitters. Maintenance of these neurotransmitters pools is strictly relying on the de novo synthesis of glutamine in astrocytes. Cerebral ischemic events disrupt the balance in uptake and re-synthesis, altering Glu, GABA, and glutamine (Gln) levels. We focused on the determining of the ratios of glutamate, GABA and glutamine in the brain of rats in the intact state, the early changes and temporal development of changes towards the recovery after disruption of balance by global cerebral ischemia. Animals underwent 15 min of global cerebral ischemia, and changes in Glu/GABA/Gln ratios in the hippocampus, cortex, and cerebellum were assessed at 3 h, 24 h, and 72 h post-reperfusion using high-resolution NMR. Ischemic preconditioning was also used to induce tolerance. In an intact rat brain, glutamate level was about twice that of glutamine in all substructures, about sevenfold compared to GABA in the hippocampus and cortex, and almost eightfold compared to GABA in the cerebellum. There were three to four times as much glutamine compared to GABA. After severe cerebral ischemia, Glu/Gln as well as GABA/Gln ratios extensively dropped in early reperfusion (3 h) and gradually increased in 72 h reperfusion time, however, only the Glu/Gln ratio recovered to the level of controls. Glu/GABA ratio remained in all three reperfusion times over the level of control animals. We observed a decrease in glutathione NMR peak in brain tissue homogenates after ischemia. The obtained data suggest the accelerated accumulation of intraparenchymal glutamate after ischemia, which was even more pronounced in the preconditioned animals three days after an ischemic event. The postischemic GABA level restoration did not achieve the level before ischemia in 72 h reperfusion, which could be one of the limiting factors in the complete postischemic GABA transmission recovery. Presented data may be of advantage not only when comparing glutamate and GABA homeostasis and neurotransmission, but also for glutamine reserve display as neurotransmitter precursor and ammonia transfer buffer in glutamate/GABA/glutamine cycle within the intact brain substructures as well after ischemic insult in rats.

## Introduction

There is a growing interest in quantifying both glutamate (Glu) and γ-aminobutyric acid (GABA) within the brain tissue, owing to their pivotal roles in complex brain function. It is widely acknowledged that these two neurotransmitters play a crucial role in maintaining the equilibrium between neuronal excitation and inhibition, which is indispensable for intricate brain complex regulatory processes. The balance among glutamate, GABA, and their precursor glutamine (Gln) is significantly perturbed by cerebral ischemic events triggering an excessive release of glutamate and GABA from respective neurons (Chen et al. 2019; Belov Kirdajova et al. 2020; Baranovicova et al. 2023).

Glutamate, the most important neurotransmitter in CNS, is a non- essential amino acid that is synthesized in neurons and glial cells. Its level in CNS is not affected by peripheral organs because it does not cross the blood–brain barrier (BBB). The average concentration of glutamate in the brain is around 12 µmol/g, in synaptic vesicles it exceeds millimolar concentration, however the glutamate level in extracellular fluids is only 0.5–2 µmol/l (ECF) (Hawkins 2009; Cooper and Jeitner 2016). The gradient of glutamate in different cerebral compartments is coordinated by specific transporters and enzymes that are responsible for its metabolism in neurons and glial cells (Robinson and Jackson 2016). In the acute phase of an ischemic event, the lack of ATP hampers the physiological function of ion pumps as the cells fail to maintain an ionic gradient (Belov Kirdajova et al. 2020), leading to excessive glutamate release from neurons as well as glia, acting as a driving force for ischemia-induced injury (Lai et al. 2014; Papazian et al. 2018; Belov Kirdajova et al. 2020). Dysfunction of clearance of glutamate leads to excitotoxicity and aberrant extrasynaptic signalling and synaptic dysfunction, and then to synaptosis, neuronal death, and behavioural alterations (Belov Kirdajova et al. 2020), and glutamate scavenging was suggested as one of the neuroprotective strategies in ischemic stroke (Kaplan-Arabaci et al. 2022).

GABA is an inhibitory neurotransmitter; it can be re-taken up by neurons or surrounding glial cells via an energy-dependent ion-based process working against a concentration gradient (Krogsgaard-Larsen et al. 2000). GABA in the glia can act as a metabolic substrate and be finally re-converted to glutamine, transferred to neurons, and converted to glutamate, which re-enters the GABA shunt. It was observed that throughout the course of cerebral ischemia/reperfusion (IR), the synthesis, release, metabolism, receptors, and transmission of GABA underwent complex pathological variations (Chen et al. 2019), making GABA a notable participant in IR injury, comprehensively reviewed by Chen et al. (Chen et al. 2019). After a cerebral ischemic event, a massive release of GABA into extracellular space was documented by many studies (Chen et al. 2019; Baranovicova et al. 2023, ). As a part of protection, both neurotransmitters, glutamate as well as GABA, are taken up by adjacent astrocytes and subsequently converted to glutamine (Chen et al. 2019; Belov Kirdajova et al. 2020). The successive release from astrocytes and uptake by neurons for use as neurotransmitter precursors is one of the most tracked processes which has been described on various levels. Besides this, the alterations in the postischemic distribution of glutamate, GABA and glutamine may be affected also due to the ischemically disordered metabolic conversion, when working as alternative metabolic and energy substrates for neurons as well as glial cells (Mierziak et al. 2021). Interestingly, the unambiguous quantitative link between the enhanced release of glutamate, GABA and glutamine to neuronal injury, post-stroke impairment, and disabilities has not been fully established (Nishizawa 2001; Dohmen et al. 2003; Chen et al. 2019; Baranovicova et al. 2023). However, as we documented in our metabolic study on the hippocampus, animals with particular protection induced by preischemia, which manifest a general decrease in neuropathological signs of ischemic injury, showed a lower extent of changes in levels of glutamate, GABA, and glutamine, and a faster metabolic recovery comparable to non-protected animals (Baranovicova et al. 2022). The protective effect of ischemic preconditioning is well known, and it was manifested in many previous studies by reduction of ischemia-reperfusion (IR) damage (Cowled and Fitridge 2020) on different levels such as a higher number of surviving neurons (Tanaka et al. 2004; Kovalska et al. 2014; Lee et al. 2020), decrease in the inflammatory response (Colàs-Campàs et al. 2020), reduction of the cellular apoptosis (Maulik et al. 1999), preservation of the energetic pool (Halestrap et al. 2007) inclusive lower extent of metabolic changes in the affected tissues (Baranovicova et al. 2021, 2022).

Remarkably, the normal neuronal synaptic function is also highly dependent on microglia and astrocytes both expressing glutamate and GABA receptors, to constantly monitor neurons and synapses (Czapski and Strosznajder 2021; Wang et al. 2022). Activated microglia, reactive gliosis and scar formation critically affect brain remodelling and neural regeneration after ischemic insult. Phagocytic astrocytes and microglia are observed after ischemic stroke to engulfe degenerating neuron debris and dead neurons at 7 days after ischemic stroke (Shi et al. 2021), whereas phagocytic microglia were mainly observed in the ischemic core region at 3 days after ischemia (Zhang et al. 2022).

One of the most important modern platforms to observe in situ metabolic conditions of an organism is NMR (nuclear magnetic resonance) spectroscopy. However, the very description of the metabolic state using NMR may sometimes run into technical limits. By employing in vivo measurements by MRS (NMR-based magnetic resonance spectroscopy) using a standard short-echo point resolved spectroscopy sequence at clinical field strength (3 T), the resonances of glutamate overlap with signals of other metabolites, particularly glutamine, leading to a great challenge in discrimination between glutamate from glutamine (Bell et al. 2021). This can be replaced, in experimental studies like this one, by high-resolution in *vitro* NMR spectroscopy, where the signals of glutamate, GABA and glutamine are well separated and can be evaluated individually.

The first part of this work focused on, so far not exactly determined, the relative ratios of Glu/GABA/Gln for rats‘ brain tissues hippocampus, cortex and cerebellum. In addition, we calculated the ratios of these metabolites to the level of N-acetyl aspartate (NAA), which is exclusively localized in neurons. The fact that the selected brain regions have unique types of neurons and thus control different neuronal processes makes the in situ brain parenchyma distribution as well as the neurotransmitter ratios functionally relevant. The obtained data may be of advantage when comparing: (i) exact glutamate and GABA homeostasis and proposed neurotransmission, (ii) glutamine reserves as neurotransmitter precursors and metabolic substrate, or also for glutamine-dependent glia activation, as well as (iii) relative metabolic aspects of the brain in situ ammonia transfer within glutamate/GABA/glutamine cycle within the brain substructures.

Even though the metabolomic approach to cerebral reperfusion injury in rodent models uses planned surgery and controlled times of the reperfusion period, relatively little is known about the progressive development of in situ metabolic responses and the role of time and prioritization in the recovery of metabolites/neurotransmitters in reperfusion period. There are particular differences in metabolic response to cerebral ischemia, where the alterations in relative levels of metabolites Glu, GABA and Gln start very early after the ischemic event while the NAA levels show a rather slow decline (Wang et al. 2001; Baranovicova et al. 2022). The objective of the second part of this work is to describe the evolution of metabolic changes systematically in three various reperfusion times in three brain substructures, which are differentially affected by global cerebral ischemia induced by four-vessel occlusion: cerebral cortex, hippocampus and cerebellum. We also focused on the effect of ischemic preconditioning/ tolerance developed after sublethal ischemic stimuli. Additionaly, we aimed to use the metabolitesratios in time-prolonged reperfusion for the description of the the prioritization of neurotransmitter level recovery, their proposed metabolic responses and we also aimetd to discuss the possibility of postischemic re-adjusting/normalisation between glutamate, GABA and glutamine in the affected brain regions.

## Materials and methods

### Induction of ischemic preconditioning and ischemia

Male Wistar rats at the age of 3.5–4 months (having a mean weight of 380 ± 26 g) were used. From a total amount of 60 rats, 41 were included for further evaluation. Complete ischemia was not achieved in 8 animals, and 11 animals died during or after surgery. Rats were kept in a temperature-controlled room at 22 ± 2^o^C on a 12-h light/dark cycle with free access to food and water. Global cerebral ischemia was induced by the four-vessel occlusion model as introduced by Pulsinelli (Pulsinelli and Buchan 1988) and already described in our previous papers (Baranovicova et al. 2018, 2020).

**Fig. 1:**
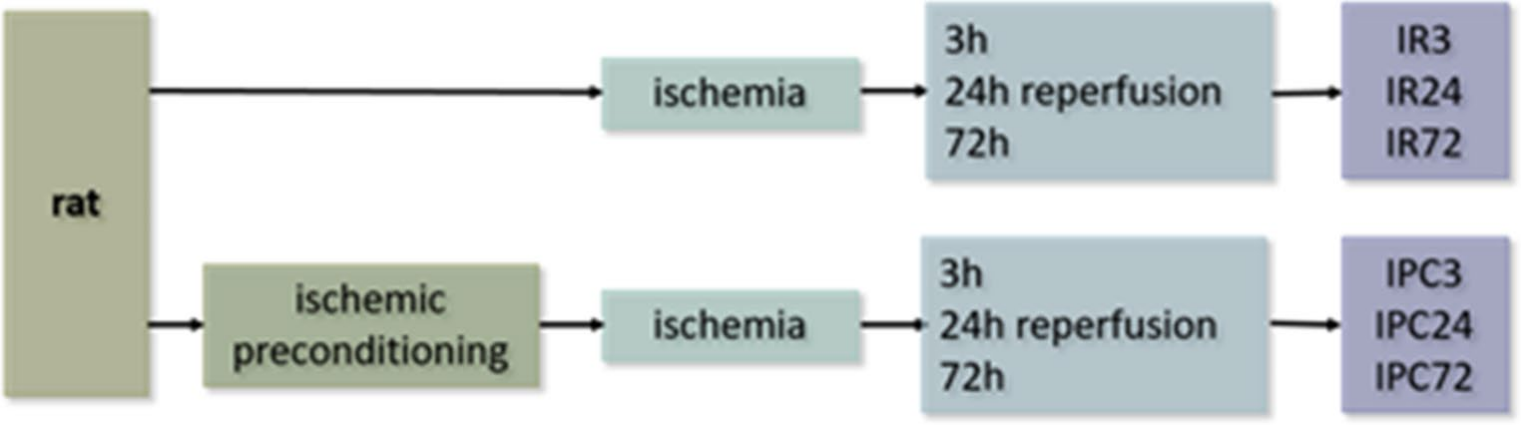
Schematic representation of operating steps in rats, IR —postischemic reperfusion, IPC — ischemically preconditioned rats following ischemia/reperfusion

On the first day, bilateral vertebral arteries were irreversibly electro-cauterized under anaesthesia with sevoflurane (a mixture of 3.5% sevoflurane in 33% O_2_ and 66% N_2_O). According to our experience, this is one of the crucial steps for the success of the 4VO model, where the improper execution of electrocauterisation can result in rupture of the vertebral arteries or cord damage leading to the loss of animal. On the second day, anaesthetized animals underwent 15 min ischemia by reversible occlusion of both common carotid arteries and exactly after 3 h, 24–72 h were sacrificed. Animals undergoing the maneuver of ischemic preconditioning were on the second day anaesthetized to induce 5 min sub-lethal ischemia and 48 h later they were subjected to 15 min ischemia. Similarly, animals were sacrificed after 3 h, 24 h, or 72 h. Operating steps and rats’ inclusion into subgroups are visualized in Fig. 1. In our previous work, we described the impact of ischemia incompleteness on metabolomic alterations in blood (Baranovicova et al. 2021). Therefore, only rats that were unresponsive lost their righting reflex and whose pupils were dilated during ischemia were selected to evaluate completed cerebral ischemia, as recommended by Pulsinelli (Pulsinelli et al. 1983). Together were included: control animals (rats without any surgical intervention, decapitated under anaesthesia) *n* = 7, rats with ischemia/reperfusion: 3 h *n* = 5, 24 h *n* = 7, 72 h *n* = 5, rats with ischemic preconditioning and ischemia/reperfusion: 3 h *n* = 5, 24 h *n* = 7, 72 h *n* = 5. All procedures on animals were performed in accordance with ethical and moral principles and approved by the State Veterinary and Food Department of the Slovak Republic.

We obtained two types of tissue specimens: (1) intact brain specimens and (2) ischemic/ reperfusion brain specimens. After decapitation, the whole brain was immediately removed while placed on ice and separated into brain subregions: (i) cerebral cortex (ii) hippocampus (iii) cerebellum, and immediately frozen and stored at −80 ^o^C. Still, in a frozen state, the tissues were homogenized using a Potter’s homogenizer (1100 rpm, 1.5 min), where the exact ratio of 5mL ice-cold ACN: H_2_O (1:1, vol.) to 1 g of tissue was kept. The homogenate was centrifuged at 4 ^o^C, 10 000 g, for 10 min. Finally, 400 µL supernatant from the hippocampus and 600 µL supernatant for the cortex and cerebellum were dried out and stored at −80 ^o^C until measurements.

### NMR data acquisition

Dried supernatants were dissolved in 500 µL D_2_O and 100 µL 0.25 M phosphate buffer (pH meter-reading 7.4) in D_2_O with TMS-d_4_ (3-(trimethylsilyl)propionic-2,2,3,3-d_4_ acid sodium salt) as an internal chemical shift reference. NMR data were acquired on 600 MHZ NMR spectrometer Bruker Avance III, equipped with a TCI cryoprobe. Sampled were after preparation stored in a Sample Jet, cooled at 5 ^o^C for the maximal time of 3 h. Before measurement, each sample was preheated to 310 K corresponding to the temperature during acquisition. An exponential noise filter was used to introduce 0.3 Hz line broadening before the Fourier transform. All data were zero-filled once. Samples were randomly ordered for acquisition.

NMR spectroscopy thanks to its robustness is a suitable method for evaluating metabolites ratios using 90-degree pulses with long enough (more than 5 s) relaxation times. Standard profiling protocols from Bruker were modified as follows: noesy with presaturation (noesygppr1d): FID size 64 k, dummy scans 4, number of scans: 128 for tissues, (cosy with presaturation (cosygpprqf): FID size 4 k, dummy scans 8, number of scans 4, spectral width 16.0125 ppm; homonuclear J- resolved (jresgpprqf): FID size 8 k, dummy scans 16, number of scans 4; profiling cpmg (cpmgpr1d, L4 = 126, d20 = 3ms): FID size 64 k, dummy scans 4, number of scans 256, spectral width 20.0156 ppm. All experiments were conducted with a relaxation delay of 5 s. The final evaluation was done on noesygppr1d-acquired data.

### Spectra evaluation

**Fig. 2:**
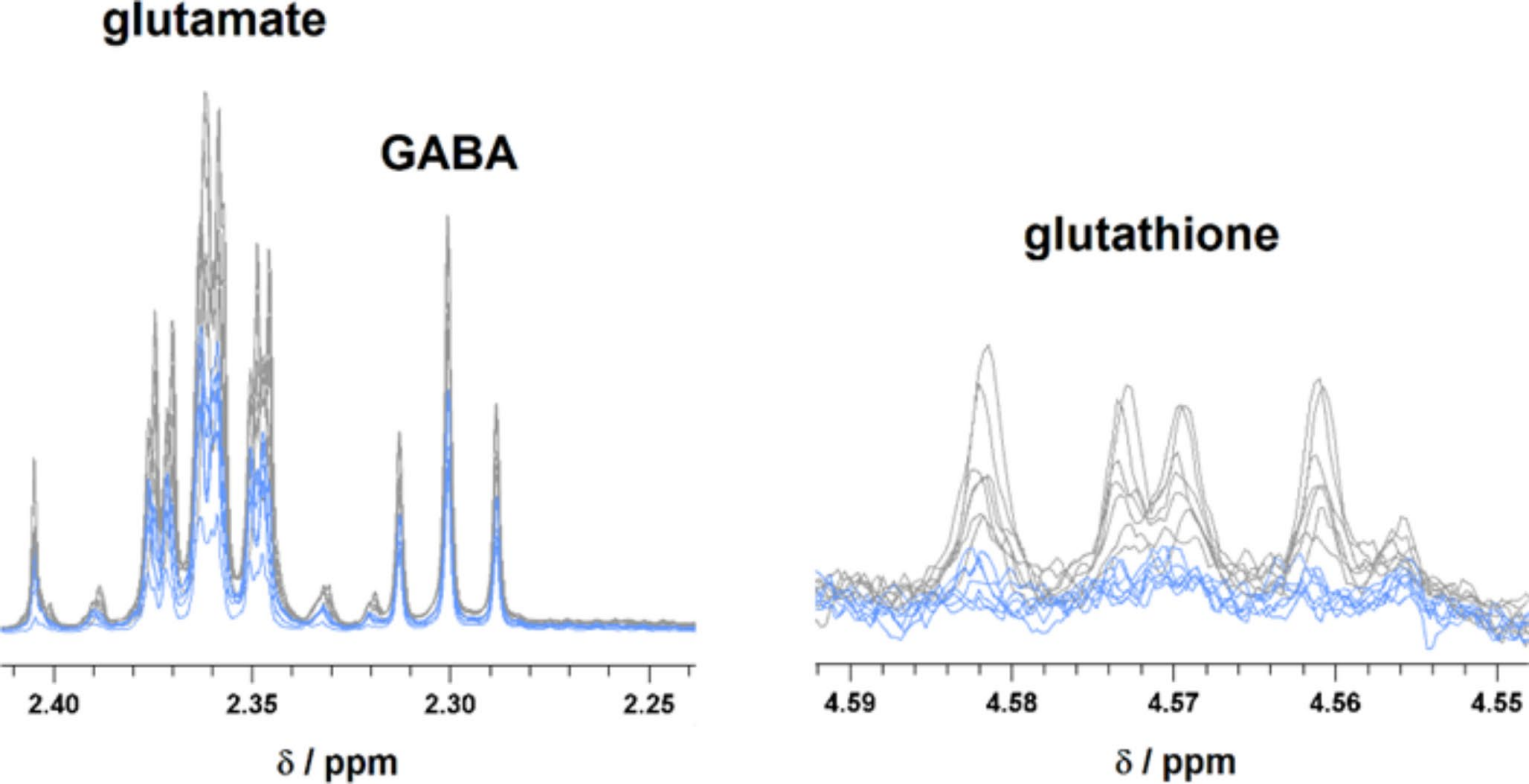
NMR signals left: glutamate and GABA, right: glutathione in hippocampal tissue extract, grey line – control animals, blue line – animals after cerebral ischemia, 24 h reperfusion

Spectra were solved using the human metabolomics database (online www.hmdb.ca, accessed in 07–09/2023) (Wishart et al. 2022), chenomx software (NMR suite 9.0), free trial version, internal metabolite database and researching metabolomics literature (Nagana Gowda et al. 2015). The peak multiplicities were confirmed in J-resolved spectra and homonuclear cross-peaks were confirmed in 2D cosy spectra. For metabolites glutamate and glutamine, a whole spectral area representing one hydrogen was integrated, for GABA and NAA spectral area representing 2, resp. 3 hydrogen was integrated and the result was divided by factor 2, resp 3. By this way, we were able to directly compare the metabolites ratios in a sample. The metabolite ratios were evaluated for every single sample and after that, the medians and IQRs were calculated for the groups. We also attempted to evaluate the levels of glutathione from the spectra. The NMR signals of glutathione were not appropriate for reliable quantification, as we demonstrate in Fig. 2 right for hippocampal tissue in controls and 24IR animals.

### Statistics

The null hypothesis of equality of population medians among controls was tested by the non-parametrical Kruskal–Wallis test with Mann Whitney U - test for pairwise comparison. The relative change was computed using the median of intensities as the difference of the medians divided by the median of the comparison group. We employed Amix v 3.9 (Bruker), Matlab 2018b, OriginPro2019 (academic licence) and SPSS software.

## Results

**Fig. 3:**
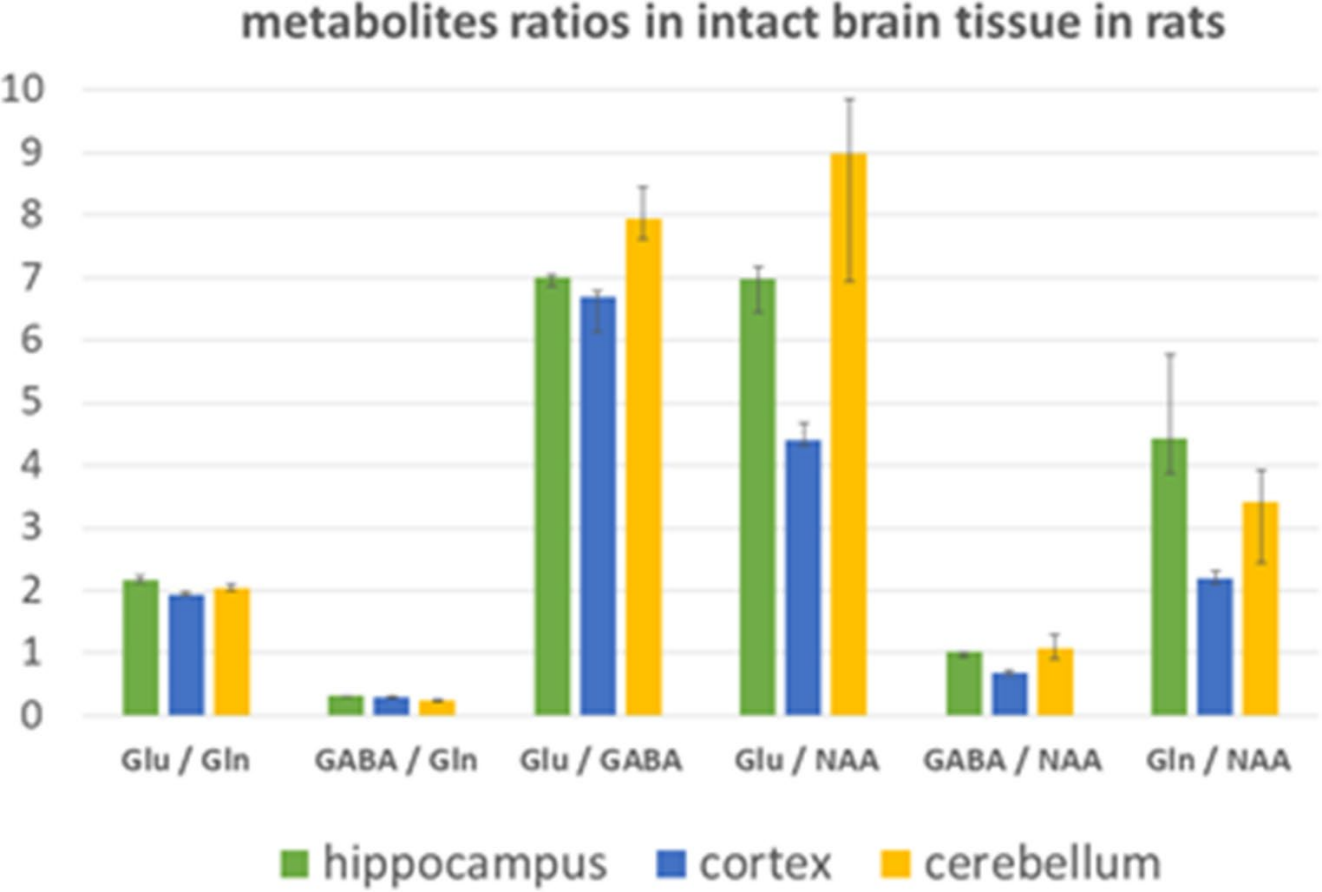
Metabolites ratios in rats’ brain structures homogenates determined by NMR spectroscopy, Glu-glutamate, Gln-glutamine, GABA - γ-aminobutyric acid, NAA- N-acetyl aspartate, data presented as medians with a range of 1st and 3rd quartiles

In our study, we determined the exact ratios of the levels of the most important metabolites in the Glu-GABA/Gln cycle in the hippocampal, cerebrocortical and cerebellar brain parenchyma. In an intact rat brain, the Glu level was about twice that of Gln in all regions, about sevenfold compared to GABA in the hippocampus and cortex, and almost eightfold compared to GABA in the cerebellum (Table 1; Fig. 3). There was three (in the hippocampus and cortex) to four times (in the cerebellum) as much Gln level compared to GABA. If we expressed levels over NAA, we found obvious differences among substructures, where the Glu/NAA ratio was almost double in the cerebellum and hippocampus against the cortex, and a similar trend was observed also for ratios of GABA/NAA and Gln/NAA. The differences in ratios of metabolites levels between rats’ brain structures: hippocampus, cortex and cerebellum were also statistically tested and manifested significant results (Table 1; Fig. 3).

**Table 1:** Statistical comparison of tmetabolites ratios in rats’ brain structures, as determined by NMR spectroscopy in vitro, Glu-glutamate, Gln-glutamine, GABA - γ-aminobutyric acid, IQR – interquartile range

	Hippocampus	Cortex	Cerebellum	Multiple comparisons	Hippocampus-cortex	Hipocampus–cerebellum	Cortex-cerebellum
	Median / IQR	Median / IQR	Median / IQR	p-value	p-value	p-value	p-value
Glu/Gln	2.16 / 0.12	1.95 / 0.05	2.05 / 0.10	*p* < 0.00005	*p* < 0.0005	*p* < 0.05	*p* < 0.005
GABA/Gln	0.32 / 0.02	0.29 / 0.03	0.25 / 0.02	*p* < 0.0001	*p* < 0.05	*p* < 0.0005	*p* < 0.001
Glu/GABA	7.01 / 0.18	6.71 / 0.66	7.95 / 0.84	*p* < 0.00005	*p* < 0.01	*p* < 0.0005	*p* < 0.0001
Glu/NAA	6.99 / 0.73	4.40 / 0.36	8.98 / 2.89	*p* < 0.00001	*p* < 0.0001	0.32	*p* < 0.0001
GABA/NAA	1.01 / 0.09	0.68 / 0.09	1.08 / 0.40	*p* < 0.0005	*p* < 0.0005	0.5	*p* < 0.001
Gln/NAA	3.05 / 0.31	2.29 / 0.20	4.41 / 1.47	*p* < 0.00005	*P* < 0.0005	0.12	*p* < 0.0001

**Fig. 4:**
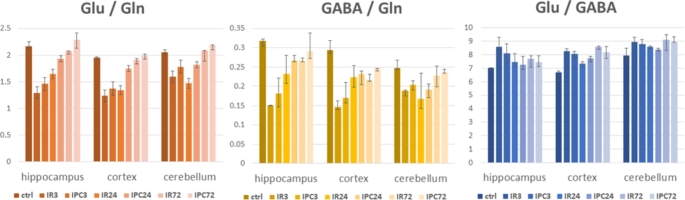
Metabolites ratios in rats’ brain tissues homogenates in an intact brain (ctrl), and after focal cerebral ischemia via four-vessel occlusion, reperfusion 3 h (IR3), 24 h (IR24) and 74 h (IR72), animals subjected to ischemic preconditioning manoeuvre before ischemia: reperfusion 3 h (IPC3), 24 h (IPC24) and 74 h (IPC72). Glu-glutamate, Gln-glutamine, GABA - γ-aminobutyric acid, data presented as medians with a range of 1st and 3rd quartiles

The global cerebral ischemia for 15 min and progressive reperfusion times differently affected all examined regions (see cerebral blood flow rate in Pulsinelli model (Pulsinelli et al. 1983). It significantly impacted the ratios of all analysed metabolites: Glu/Gln, GABA/Gln and also Glu/GABA. As expected from data from of CBF rate (Pulsinelli et al. 1983), Glu/Gln dropped noticeably during early reperfusion (3 h) and gradually increased over 24 h reperfusion in the hippocampus and cortex, but not the cerebellum. In 72 h reperfusion, Glu/Gln ratio was approaching the level of controls in all three substructures. This was not detected for the GABA/Gln ratio which, after an initial decrease in 3 h reperfusion, increased towards, but not achieving the level of control in the hippocampus in 72 h reperfusion. In the cerebellum, after a decrease in 3 h reperfusion, the minimal value of GABA/Gln ratio could be observed in 24 h reperfusion, with further increase in 72 h reperfusion, however, still obviously remained under the level of controls. In the cortex, the values after an initial decrease were very close to each other in 24 h and 72 h reperfusion also under control levels. In all three substructures, the GABA/Gln level remained below the level of non-ischemic animals in 72 h reperfusion. The last evaluated ratio, Glu/GABA was in all three reperfusion times in all brain subregions over the level of control animals, not showing an obvious trend to increase or decrease with reperfusion (Table 2; Fig. 4). Finally, based on visual inspection of the spectra, we could conclude a decrease in glutathione in the tissue after cerebral ischemia (Fig. 2).

In animals subjected to ischemic preconditioning prior to ischemic insult, we observed an effect of the proposed development of ischemic tolerance. The ratio of Glu/Gln and GABA/Gln were less affected by ischemia in all three substructures in all three reperfusion times, with values (in comparison to non-preconditioned animals) closer to those found in controls. This was, although to minimal difference, also true in almost all reperfusion times for Glu/GABA ratio (except cortex 24 h IR-IPC comparison) (Table 2; Fig. 4).

**Table 2:** Metabolites ratios in rats’ brain structures, as determined by NMR spectroscopy in vitro in three reperfusion times 3 h – IR3, 24 h – IR24,72 h – IR72, animals with ischemic tolerance earned by ischemic preconditioning in three reperfusion times 3 h – IPC3, 24 h – IPC24,72 h – IPC72, ctrl – non-ischemic animals, * significant change against ctrl (p-value < 0.05), Glu-glutamate, Gln-glutamine, GABA - γ-aminobutyric acid

		Hippocampus		Cortex		Cerebellum	
		Median	IQR	Median	IQR	Median	IQR
ctrl	Glu/Gln	2.16052	0.11565	1.94734	0.04948	2.05282	0.09652
IR3	Glu/Gln	1.29195*	0.04637	1.23663*	0.02776	1.59488*	0.05369
IPC3	Glu/Gln	1.45979*	0.40306	1.37317*	0.60455	1.78337*	0.28634
IR24	Glu/Gln	1.64769*	0.3997	1.34372*	0.30153	1.46953*	0.64177
IPC24	Glu/Gln	1.93401*	0.0772	1.74984*	0.16968	1.82154*	0.17758
IR72	Glu/Gln	2.06936*	0.0619	1.91224	0.10558	2.07999	0.20319
IPC72	Glu/Gln	2.28241	0.28389	1.99186	0.09434	2.18118	0.09656
ctrl	GABA/Gln	0.31841	0.01827	0.29358	0.03195	0.24783	0.02306
IR3	GABA/Gln	0.15068*	0.00102	0.14656*	0.02279	0.18803*	0.01494
IPC3	GABA/Gln	0.18201*	0.07436	0.17028*	0.05411	0.20381*	0.02397
IR24	GABA/Gln	0.2315*	0.07271	0.22296*	0.05626	0.16674*	0.09111
IPC24	GABA/Gln	0.26748*	0.01646	0.23238*	0.03539	0.19861*	0.02575
IR72	GABA/Gln	0.2663*	0.01015	0.21589*	0.01857	0.22847*	0.05266
IPC72	GABA/Gln	0.29106	0.06659	0.24593*	0.00639	0.23553*	0.00945
ctrl	Glu/GABA	7.01283	0.18193	6.70706	0.66333	7.94953	0.83979
IR3	Glu/GABA	8.58457*	0.14935	8.25645*	0.57035	8.92745*	0.3446
IPC3	Glu/GABA	8.10734*	1.44673	8.06483*	0.40218	8.77303*	0.40185
IR24	Glu/GABA	7.4553*	0.99677	7.32265*	0.41565	8.5754*	0.99076
IPC24	Glu/GABA	7.26542	0.78193	7.71686*	0.89793	8.3884*	0.7714
IR72	Glu/GABA	7.67546*	0.81727	8.5738*	0.27028	9.10409*	1.1793
IPC72	Glu/GABA	7.44288	0.82072	8.17203*	0.8848	8.95203*	0.42297

## Discussion

### Intact brain

Our analytical approach using high-resolution in vitro NMR spectroscopy study confirmed that glutamate, which acts as an excitatory neurotransmitter and important metabolic and nitrogen metabolite, is highly concentrated in all intact brain regions. We found the level of glutamate as about twice that of glutamine in rats’ brain substructures, and significant differences were indicated also between the hippocampus, cerebellum and cortex (Table 1). Inhibitory neurotransmitter GABA (Cooper and Jeitner 2016) showed around seven to eight times smaller levels in brain substructures compared to glutamate, and, likewise, the ratios significantly differed among individual brain regions. The recent literature data provides similar results on methanol/water extract from the brain hippocampal homogenate using an alternative high-performance/pressure liquid chromatography (HPLC) (Zieminska et al. 2018), which indicates the applicability of both methodologies (in vitro NMR as well as HPLC) for determination of neurotransmitters levels in brain tissue homogenates. It should be also mentioned that the method of calculation can slightly affect the results, where the median of the ratios (as used in our work) does not have to be the same as the ratio of the medians, or the ratio of the average values.

The variability in metabolite abundance among brain structures could be linked to the different densities of respective neurotransmitter receptors, specific metabolic enzymes and the general role of metabolites in the neuronal and glial cell types within the brain structures. In the previous work, Duarte et al. showed a relatively strong correlation between regional glutamate and NAA (which is believed to be localized exclusively in neurons (Moffett et al. 2007) levels across mouse age and gender, presenting NAA and glutamate as relevant markers for neuronal density or function (Duarte et al. 2012). We calculated ratios of both neurotransmitter levels and glutamine to NAA. In our study, the ratios Glu/NAA, GABA/NAA, as well as Gln/NAA increased in line: cortex, hippocampus, and cerebellum. If we use data from mouse neuronal density, the order in which the neural density increases (Keller et al. 2018) decreased the values of ratios of metabolites with NAA value in the denominator (Table 2; Fig. 3). However, from this comparison, we can conclude rather the relation of neural density to the level of NAA than to the amino acids (neurotransmitters) levels.

The fact that the ratios between metabolites Glu/Gln and GABA/Gln cannot be linked with the neural densities, (when compared with the data from mice (Keller et al. 2018), is based likely on the multiple metabolic functions of glutamate, GABA and glutamine among neurons, astrocytes and microglia (microglia represents 5–12% of all glial cells (Lawson et al. 1990) in the brain substructures. Glutamate is stored mainly in neuronal vesicles prepared for its neurotransmitter function, however, a significant proportion of glutamate is oxidatively metabolized by conversion to oxoglutarate to the tricarboxylic acid cycle (TCA) in neurons (Hassel 2001), as well as glial cells (Gondáš et al. 2023), and also used as a substrate of glutamine synthetase to detoxify ammonia and produce glutamine (Schousboe et al. 2014). The main source of brain parenchymal GABA are GABAergic neurons, however, a substantial GABA synthesis also occurs in glial cells (Angulo et al. 2008; Héja et al. 2012; Serrano-Regal et al. 2020). Maintaining the availability of both neurotransmitters in brain tissues, their mutual conversion with glutamine is secured by constantly ongoing Glu-GABA/Gln shuttle between neurons and astrocytes, a pathway resulting in about 85% of the glutamine synthesis in rat cortex (Rothman et al. 2011). It was presented that approximately 30% of total glutamate entering astrocytes is subjected to oxidative processes and 70% contributes to the Glu-Gln cycle, which indicates that biosynthesis of glutamate continuously occurs in the neurons, supplying nerve terminals with sufficient neurotransmitters (Nieoullon 2009). The carbon skeleton of glutamate via entering the Krebs cycle is converted to malate, which is ultimately turned to pyruvate by the malic enzyme (ME). As an alternative, pyruvate kinase (PK) and phosphoenolpyruvate carboxykinase (PEPCK) work together to convert oxaloacetate (OAA) to pyruvate. Interestingly, for the complete oxidation of glutamate, all enzymes must operate in the direction to produce pyruvate, which is a substrate of pyruvate dehydrogenase that re/enters the Krebs cycle and is entirely oxidized to CO_2_.

The distribution of Glu and GABA over brain regions is surely linked with a proportion between excitatory glutamatergic and inhibitory GABAergic neurons. In intact rat brains, the cerebellum showed almost 20% higher Glu/GABA ratio compared to the cortex, and the hippocampus manifested almost 30% higher proportion of GABA to glutamine compared to the cerebellum and about 10% higher Glu/Gln ratio than the cortex (Table 1). Quantitative immunohistochemistry studies and genetic markers have estimated the ratio of approximately 4 to 1 of glutamatergic neurons for every GABAergic neurons in the cortex (Swanson and Maffei 2019), and it is even generally accepted that 20%of all neurons in rodents’ cortex are GABAergic interneurons (Sahara et al. 2012). The literature provides also data about the hippocampal circuit of GABAergic inhibitory interneurons which represents 10–15% of the total neuronal population (Pelkey et al. 2017). These data, showing a higher portion of glutamatergic to GABAergic neurons in the hippocampus than in the cortex could be indicative for the explanation of higher Glu/GABA levels in the hippocampus than in the cortex. In general, the determination of precise numbers of GABAergic and glutamatergic neurons in brain structures faces generally several methodological problems due to the complexity of the brain’s regional cellular and layer composition. Moreover, this number is influenced by genetic factors, developmental changes and as result of time-dependent neural plasticity changes. As detected in mice and also in other species, the levels of glutamate and GABA in the hippocampus and cortex show an age-dependent decline (Duarte et al. 2012). Unfortunately, due to methodological constraints, there is a persisting lack of detailed data about the exact quantities of glutamate and GABA pools in brain substructures, their precise cellular localization and the contributions of non-neuronal cells to total brain regional glutamate and GABA levels. The quantitative data may shed deeper light on aspects such as the availability of glutamine as a precursor for neurotransmitters, its role as a metabolic substrate, and nitrogen carrier or its involvement in the activation/proliferation of glial cells dependent on glutamine. Additionally, knowing the exact level of glutamine could be valuable in understanding the dynamics of ammonia transfer within the glutamate, GABA, and glutamine cycle and other amino acid metabolism.

### Ischemic brain

Our work provides the first comprehensive study to identify and measure levels of the most important amino acids acting as excitatory and inhibitory neurotransmitters in the brain region parenchyma. As presented by Pulsinelly et al., who introduced the animal model of global cerebral ischemia also used in this study (Pulsinelli and Buchan 1988), the regional cerebral blood flow, as well as glucose metabolism in the brain regions, are differently affected by severe but transient global ischemia (Pulsinelli et al. 1983). These are the first original data which utilizes a reliable and novel methodological approach for the quantification of amino acid levels in brain homogenates.

Brain tissue extracts in 3 h reperfusion after the ischemic event were characterized by a generally decreased glutamate/glutamine ratio, which was most pronounced in the hippocampus–of about 40%, then in the cortex of about 35% and the smallest change was observed in the cerebellum, of about 20% (Fig. 3; Table 2). This is in line with the results of blood flow rate measurements in the 4VO model of global ischemia (Pulsinelli et al. 1983), where brain regions exposed to severe ischemia such as the hippocampus and cortex showed greater metabolic alterations than the cerebellum. Observed changes are paralleled with the excessive glutamate release from neurons and glial cells, (Belov Kirdajova et al. 2020) which is a cell death mechanism known as glutamate excitotoxicity, and is considered one of the major causes of damage after the ischemic event in the brain (Choi 2020). Brain glutamine synthetase, localized in all cells of the astroglial family (Anlauf and Derouiche 2013), increases after the ischemic event (Petito et al. 1992), and extracellular glutamate is converted to glutamine. As our results indicate, within three days of reperfusion (IR72), the ratio glutamate/glutamine is able to almost recover to the values observed for non-ischemic tissue (Fig. 4). Moreover, it is important to note, that both the ratio as well as the relative aminoacids pools themselves are almost recovered to the preischemic level, as we have shown in the previous work on the hippocampus (Baranovicova et al. 2022) and cortex (Baranovicova et al. 2021).

**Fig. 5:**
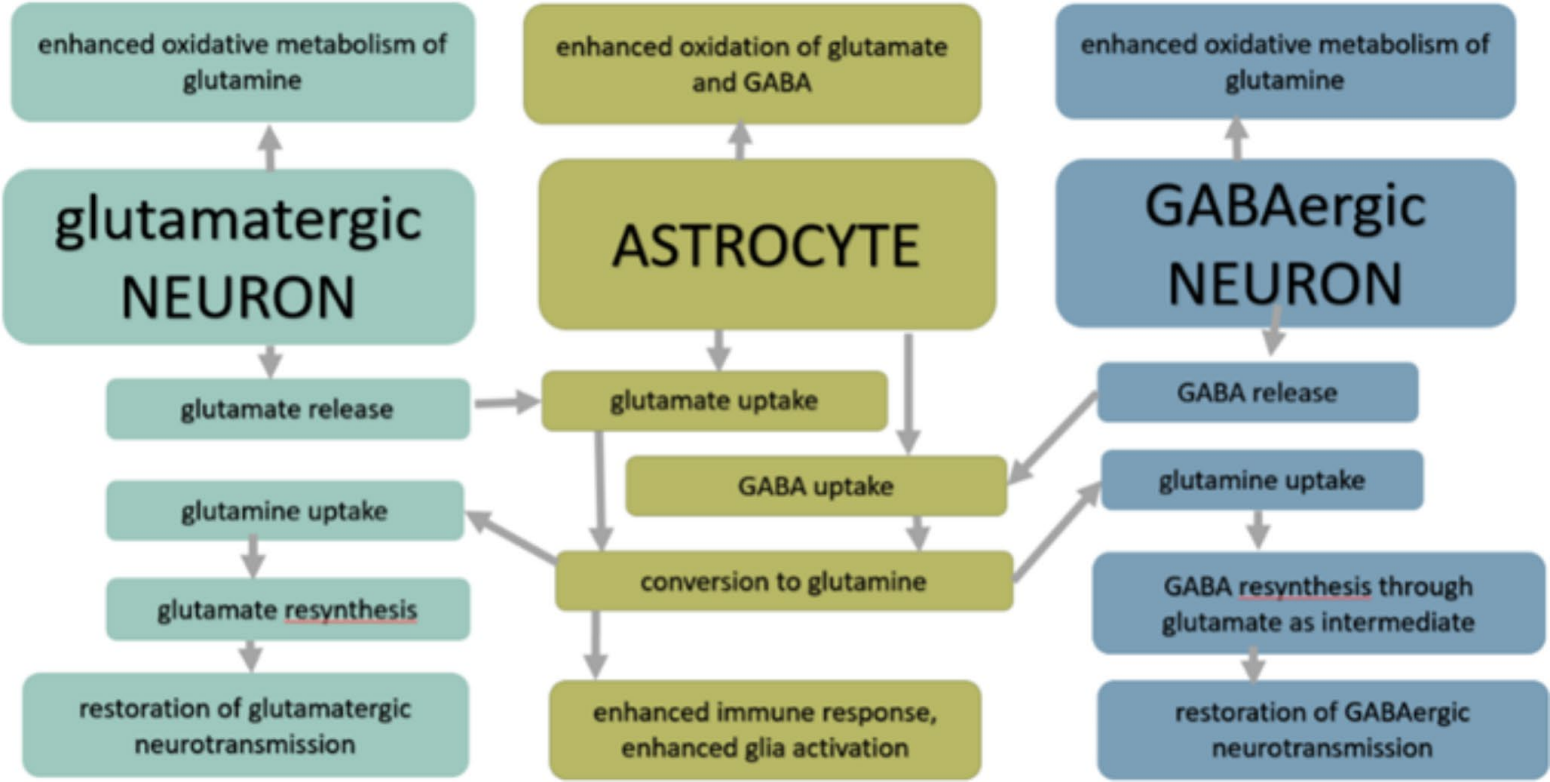
Simplified turnover of glutamate/GABA and glutamine, enhanced after ischemia-induced excessive neurotransmitter release, with proposed postischemically accelerated metabolic oxidation in the reperfusion/recirculation period

The results by Globus et al. (Globus et al. 1988) suggest that not the quantitative release of glutamate by itself accounts for the pattern of selective vulnerability of the organs, but, instead, the imbalance between excitation and inhibition may play a major role in the mediation of neuronal damage (Globus et al. 1991). In fact, we observed a decrease in GABA/Gln ratio for about 60% 3 h after ischemia (3IR) in the hippocampus, then in the cortex of about 50%, and the lowest change was detected in the cerebellum, of about 25%, which correlates with the proportion of observed compromised blood flow rate in 4VO model (Pulsinelli et al. 1983). These ratios did not fully recover in 72 h reperfusion after the ischemic event (72IR), remaining at the portion of 83% for the hippocampus, 75% for the cortex and 92% for cerebellum related to those found in the intact brain. Throughout cerebral I/R, the inhibitory neurotransmitter GABA undergoes complex pathological variations (Chen et al. 2019). GABA is, similarly to glutamate, after cerebral ischemia, released from neurons and also microglia, and either reabsorbed by neurons or uptaken by surrounding glial cells for further metabolism (Fig. 5). To compare the metabolic prioritization in restoring levels of glutamate and GABA after ischemia, we calculated the Glu/GABA ratio, which was increased over the first 72 h reperfusion time in all ischemic brain substructures (Fig. 4). Not only in tissue homogenates, as used in our study, including a contribution of both, extracellular and intracellular components, but increased Glu/GABA ratio was also observed by HPLC when monitoring neurotransmitters in interstitial fluid by analyzing microdialysate by Zeng et al., in rats after middle cerebral artery occlusion (MCAO) in the first 130 minutes after onset of ischemia (Zeng et al. 2007) as well as by Wang et al. in 90 min reperfusion after MCAO in mice (Wang et al. 2001). The results of this study and results of our previous works (Baranovicova et al. 2021, 2022) indicate the recovery of the relative glutamate, but not GABA (Baranovicova et al. 2022) pools in brain tissues in 72 h reperfusion. The Glu/GABA ratio in hippocampal tissue in 72 reperfusion time found in this study suggests that the ischemic balance between glutamate and GABA will be established later than 72 h after an ischemic event.

It is interesting to note that despite the postischemic damage or even death of neurons, a large part of the decreased glutamate amount seen after the ischemic event in the brain is replaced in the 72-hour reperfusion period. This occurs relatively quickly in comparison to the re-synthesis of the neuron-characteristic metabolite N-acetyl aspartate (NAA), considered to be an indicator of neural health and fitness (Demougeot et al. 2004). NAA levels were observed to elevate only 8 days after ischemia and continued to increase until 30 days (Demougeot et al. 2004). It can be assumed that the intact, not damaged, neurons and glia would efficiently synthesize glutamate to substitute its deprivation due to an ischemic event and therefore compensate for the altered synthetic function of damaged neural cells. On the other hand, not fully recovered levels of GABA three days after ischemia, may be the reason for the persistently limited feasibility of reestablishing GABAergic transmission after ischemia. Reduced GABA transmission with increased glutamate signals occurring in cerebral I/R injury was documented in previous studies (Chen et al. 2019), which also correlates with the increased glutamate/GABA ratio in affected brain regions after ischemia. In this context, an enhancement of the GABA actions and decreased glutamate can be a potential preventive strategy for cerebral I/R injury, which may be achieved by different enhancers of GABA transmission that could partly restore the balance in GABA and glutamate transmissions (Chen et al. 2019). GABA also affects functions of astroglial and microglial cells as well as peripheral immune cell populations accumulating in the ischemic territory and brain regions remote to the lesion (Michalettos and Ruscher 2022), however, the complex interactions between inflammatory cascades and neuronal functions are still not comprehensively understood.

A crucial role in cell defence against oxidative stress and the disruption of Zn^2+^ homeostasis plays glutathione, which is the most abundant low molecular weight thiol compound in the brain. Glutathione is an antioxidant with cellular protective functions, including reactive oxygen species scavenging in the brain, and it was found to preserve the disruption of BBB after ischemic injury and improve the survival of brain endothelial cells (Song et al. 2015). Decreased levels of glutathione after transient ischemia in the hippocampus were described previously (Higashi et al. 2021), and similarly, we observed decreased intensity of glutathione peaks in NMR spectra in brain tissue homogenates after cerebral ischemia, however, the quantitative evaluation of the NMR signals were not reliable. As just glutamate is an essential part of glutathione synthesis, the disturbances in glutamate level after cerebral ischemia may unfavourably affect glutathione synthesis and therewith also suppress its function of redox protection.

In addition to complex damaging glutamate excitotoxic mechanisms, excitotoxic glutamate inhibits mTOR signalling and causes elevated neuronal insulin resistance (Pomytkin et al. 2019), making neurons more vulnerable to metabolic stress induced by the ischemic event. On the other hand, this would be a sign of metabolic demand alternations to use alternative energy substrates to preserve cell viability. Under physiological conditions, the use of glutamate and GABA by astrocytes and glutamine by neurons as useful and actual energy substrates is not essential. However, after ischemia, when glucose oxidative metabolism in the brain is compromised, the metabolic conversion of these amino acids as alternative energy substrates was observed to increase (Pascual et al. 1998). It seems that cells in/after oxygen restriction might alternatively use molecules with primary other functions such as neurotransmitters and/or alternative energy substrates. As a consequence, this can further shift the balance between glutamate, GABA and glutamine in the affected brain parenchyma.

As documented above, the studies on tissue homogenates via in vitro metabolomic methods cannot reproducibly discriminate the distribution of glutamine among neurons, astrocytes and microglia, verify its presence in glial cells reuptake by neurons for neurotransmitter re-synthesis. When translating our study performed on animal models of the global cerebral ischemia, it should be also considered that the extent of the metabolic response may change depending on ischemia severity, as well as the postischemic profiles of glutamate and GABA vary in core versus peripheral zones (Wang et al. 2001).

### Rats with ischemic protection earned by ischemic preconditioning (IPC)

The alteration in metabolic response in the animals with induced ischemic tolerance/neuro-protection was already shown by Dave et al., which found a higher GABA extracellular concentration within 80 min reperfusion in IPC animals, as a consequence of proposed neuroprotective preconditioning (Dave et al. 2005). In our work, animals subjected to ischemic preconditioning before ischemia showed, that all tested parameters manifested smaller postischemic responses compared to animals with single severe cerebral ischemia (Fig. 4). This weaker metabolic response in the levels of Glu/GABA/Gln in IPC animals provides further evidence for documenting the protective effect of sub-lethal ischemia to ischemic injury/damage. In this context, we would like to stress an exceeding Glu/Gln ratio compared to control animals at prolonged 72 h reperfusion time in IPC animals, which suggests accelerated glutamate metabolism recovery. The higher tissue glutamate level supports both the glutamatergic neurotransmission and also GABA resynthesis and other processes such as e.g. immunomodulatory actions of GABA (Michalettos and Ruscher 2022), and it could be considered as one of the important metabolic features in postischemic recovery.

## Conclusion

In an intact rat brain, we determined the amount of glutamate as 7–8 fold higher relative to GABA and about two-fold relative to glutamine level with subtle variations in brain regions: hippocampus, cortex and cerebellum. Severe global cerebral ischemic event remarkably disturbed the balance among these metabolites in correlation with brain regional dispirited perfusion rate restriction and caused a decrease in the Glu/Gln and GABA/ Gln ratios with the tendency to partial gradual recovery over 72 h reperfusion in all analyzed regions. The putatively uniform postischemic ratio Glu/GABA in longer reperfusion time with values over the level of controls signalizes accelerated postischemic glutamate metabolism recovery with consequent conversion to GABA. In ischemic animals, we observed decreased amount of glutathione, suggesting decreased antioxidant potency of the tissue in ischemia/ reperfusion. The response to cerebral ischemia was proportional to the blood perfusion rate: the most pronounced in the hippocampus, followed by the cortex and the lowest extent of metabolic changes were observed in the cerebellum. Animals which underwent the manoeuvre of ischemic preconditioning showed significantly lower degrees of changes in the levels of neurotransmitter/ (amino acids), supporting the induction of the proposed protective tolerance effect.

Metabolic response in the late 72 h reperfusion time suggests that neural cells accelerate glutamate metabolic recovery to substitute the deprivation induced by ischemic event and presumably compensate for the altered synthetic function of damaged neural cells. Level of GABA not fully recovered in the late 72 h reperfusion time in all monitored brain areas indicating still limited conditions for proper GABA neurotransmission and connectivity. Based on our results, to establish the control balance in the neurotransmitter levels after severe global ischemia/reperfusion, the additives supporting the ischemic glutamate/GABA ratio conversion could play a substantial role in the proposed alternative metabolic treatment of ischemic event.

## Data Availability

No datasets were generated or analysed during the current study.
